# MicroRNAs and genes regulating responses to hypoxia and inflammation expression levels in blood leukocytes as potential biomarkers of initial oxygen deficiency tolerance

**DOI:** 10.3389/fmolb.2026.1796967

**Published:** 2026-04-01

**Authors:** Maria Kirillova, Dzhuliia Dzhalilova, Margarita Maiak, Vladimir Kirillov, Ivan Tsvetkov, Nikolai Fokichev, Olga Makarova

**Affiliations:** 1 Laboratory of Inflammation Immunomorphology, Avtsyn Research Institute of Human Morphology of Federal State Budgetary Scientific Institution, Petrovsky National Research Centre of Surgery, Moscow, Russia; 2 Department of Histology, Petrovsky Medical University, Moscow, Russia; 3 Laboratory of Stem Cell Genetics, Research Centre for Medical Genetics, Moscow, Russia; 4 Laboratory of Molecular Genetics, National Medical Research Center for Obstetrics, Gynecology and Perinatology named after Academician V. I. Kulakov of the Ministry of Health of the Russian Federation, Moscow, Russia; 5 Faculty of Biology and Biotechnology, HSE University, Moscow, Russia

**Keywords:** biomarkers, hypoxia-inducible factor, hypoxia tolerance, hypoxic exposure, microRNA

## Abstract

**Introduction:**

Hypoxia-inducible factors (HIFs) play central roles in evoking responses to hypoxia, and their activities are regulated by numerous molecules, including microRNAs. Organisms typically show differing tolerances to oxygen deficiency; the most common method of determining such tolerance involves examining the effects of sublethal hypoxic exposure (SHE) in a decompression chamber, which can lead to pathological changes in the internal organs. The aim of the present study was to investigate the microRNAs and genes regulating cellular responses to hypoxia and inflammation expression levels in the peripheral blood leukocytes of laboratory animals before and 1 month following determination of hypoxia tolerance.

**Methods:**

In animals not exposed to hypoxic exposure, blood was collected from the tail vein. One month later, the rats’ resistance to hypoxia was determined at a critical altitude (11,500 m) in a single test in a decompression chamber, based on the “gasping time” before assuming a lateral position and the appearance of signs of asphyxia. Two groups of rats were identified – tolerant and susceptible to hypoxia. The expression of *mRNA Hif1a*, *Epas1*, *Hif3a*, *Arnt*, *Vegf*, *Epo*, *Egln1*, *Nfkb*, *Il1b*, *Tnfa*, *Tgfb* and microRNA *rno-miR-210-5p*, *rno-miR-210-3p*, *rno-miR-107-5p*, *rno-miR-107-3p*, *rno-miR-145-5p*, *rno-miR-145-3p*, *rno-miR-155-5p*, *rno-miR-155-3p* in leukocytes was determined by real-time PCR. To assess the impact of SHE on internal organs, morphological and morphometric studies of the lungs were carried out.

**Results:**

Compared to tolerant rats, the susceptible animals demonstrated an initial high proinflammatory potential characterized by high *Hif1a*, *Epas1*, *Hif3a*, and *Nfkb* expression along with low levels of *rno-miR-155-3p* and *rno-miR-210-3p* in the leukocytes. We observed the activation of proinflammatory responses in both tolerant and susceptible animals, and additional expression level increases of *Il1b* and *Tnfa* were noted only in the susceptible rats.

**Discussion:**

SHE has extended effects on organisms that last for at least a month. The indicators identified herein, which differ between the hypoxia-tolerant and hypoxia-susceptible animals before sublethal hypoxic exposure, can therefore be considered as biomarkers of the initial hypoxia tolerance potential.

## 1 Introduction

Systemic hypoxia accompanied by impaired oxygen absorption or transport typically occurs in critical conditions, such as chronic cardiovascular failure, chronic inflammatory lung diseases, and anemia (Lee et al., 2019). Furthermore, hypoxia increases the likelihood of complications and mortality in severe infectious and inflammatory diseases, sepsis, and acute respiratory distress syndrome, similar to the conditions developed for the COVID-19 infection (Urakov et al., 2022).

In such cases, the hypoxia-inducible factors (HIFs) are the main transcription factors responsible for evoking responses to hypoxia. Currently, three HIF-α isoforms have been characterized in mammals, namely HIF-1α, HIF-2α, and HIF-3α, along with the HIF-β protein (Semenza, 2012; Duan, 2016; Ravenna et al., 2016; Serocki et al., 2018; Burtscher et al., 2021). The activities of the oxygen-dependent HIF-α subunits are mainly regulated through hydroxylation by prolyl hydroxylases (PHDs); beyond this canonical pathway, there exist additional regulatory mechanisms, including RNA interference involving microRNAs (Leaman et al., 2005).

The main regulator of cellular response to oxygen deficiency is miR-210, which has over 10 identified mRNA targets that are involved in angiogenesis, differentiation, and mitochondrial oxygen consumption (Fasanaro et al., 2008; Mizuno et al., 2009; Chan et al., 2009). There are other microRNAs that can suppress the translation of the HIF family: miR-155 targeting HIF-1α (Yang et al., 2016), miR-107 targeting HIF-1β (Yamakuchi et al., 2010; Deng et al., 2016), and miR-145 targeting HIF-2α (Zhang et al., 2014).

The individual tolerances to hypoxia can influence the severity of inflammatory processes and depend on factors such as sex, age, biorhythms, and comorbidities (Esper et al., 2006; Mayr et al., 2014; Kosyreva et al., 2019; Dzhalilova et al., 2019). Humans and laboratory animals are known to differ in their hypoxia tolerances as well as several parameters, namely the levels of *Hif1a* expression, erythropoietin, corticosterone, and antioxidant enzymes, among others (Lukyanova et al., 2009; Ghosh et al., 2012; Padhy et al., 2013; Jain et al., 2013; 2014; Dzhalilova and Makarova, 2020). Currently, human tolerance to oxygen deficiency is determined by exposing the subjects to an altitude corresponding to several thousand meters (up to 6,500 m), especially when selecting individuals for extreme professions (e.g., pilots, astronauts) (Bustamante-Sánchez et al., 2019; Tu et al., 2020; Lu et al., 2018; Kammerer et al., 2018). Since laboratory animals are generally more tolerant to hypoxia than humans, extremely high altitudes are used to determine their resistance to oxygen deficiency compared to humans. This critical “altitude” is 10,000 m for C57Bl/6 mice (Vedunova M.V. et al., 2014) and 11,500 m for Wistar rats (Kirova et al., 2013). Animals are classified as tolerant or susceptible based on the “gasping time,” which is the duration of exposure at a given “altitude” before assuming a lateral position and showing signs of asphyxia. With this assessment method, a sublethal hypoxic exposure (SHE) is used as a rule, which can lead to pathological and inflammatory changes in the internal organs (Makarova et al., 1992). Therefore, there is a necessity to develop fast and accurate methods for hypoxia tolerance assessment; one such method involves determining the expression of mRNAs and microRNAs that are known to regulate cellular responses to oxygen deficiency in the peripheral blood leukocytes.

The aim of the present work was to investigate the expression levels of microRNAs and genes regulating cellular responses to hypoxia and inflammation in the peripheral blood leukocytes of laboratory animals before and after hypoxia tolerance determination.

## 2 Materials and methods

### 2.1 Animals

The study was conducted on male Wistar rats aged 2–3 months (n = 30) and weighing 250–300 g that were obtained from the Branch of the Federal State Budgetary Institution of Science, State Scientific Center of the Russian Federation, Institute of Bioorganic Chemistry named after Academicians M. M. Shemyakin and Yu. A. Ovchinnikov of the Russian Academy of Sciences (Branch of the State Scientific Center IBCh RAS). The experimental unit was a single animal. The rats were housed at five per 48 cm × 37.5 cm × 21 cm cage having a footprint of 1,500 cm^2^ (Tecniplast, Italy) that were designed to hold up to eight rats weighing 200–300 g or less at a regulated room temperature of 25 °C ± 2 °C under 12:12 h light–dark cycle and 40%–50% relative humidity with unlimited access to water and food (Char; JSC Range-Agro, Turakovo, Russia). When working with the experimental animals, we were guided by the principles of the European Convention for the Protection of Vertebrate Animals used for Experimental Purposes (Strasbourg, 1986) and Directive 2010/63/EU of the European Parliament and the Council of the EU. The study protocols were approved by the Local Ethics Committee of Petrovsky National Research Center of Surgery, Moscow, Russia (protocol no. 11 dated 20 December 2024). All procedures were in accordance with the Animal Research Reporting of *In Vivo* Experiments (ARRIVE) guidelines. According to literature, an animal’s hypoxia tolerance depends on its sex and age; it has been demonstrated that hypoxia-tolerant organisms are predominantly females, whereas susceptible and normal organisms are predominantly males, with sex-based differences in the morphofunctional state of the immune system (Kosyreva et al., 2020; Dzhalilova et al., 2020). In addition, it was demonstrated that newborn animals are the most tolerant to hypoxia while prepubertal animals are the least tolerant (Dzhalilova et al., 2021). To equalize sex- and age-based differences, this study was performed only with male rats. All methods were performed in accordance with the relevant guidelines and regulations.

### 2.2 Hypoxia tolerance determination and SHE modeling

**FIGURE 1:**
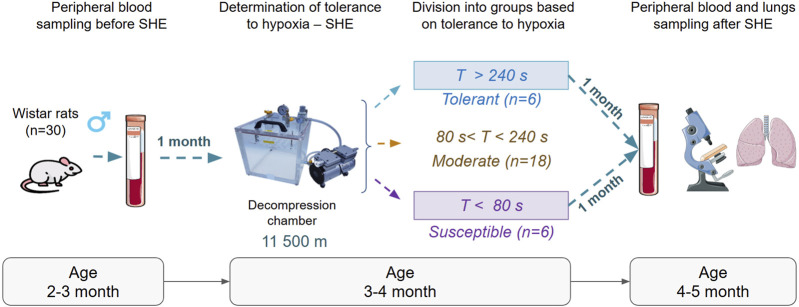
Schematic of the experimental procedures in this study.

The experimental plan implemented in this study is shown in Figure 1. In the case of animals that were not subjected to hypoxic stress, we collected 1 mL of blood from the tail vein into tubes containing K3-EDTA (Greiner Bio-One) under intramuscular Zoletil anesthesia (2 mg/kg; Virbac Sante Animale); then, the erythrocytes were lysed and IntactRNA (Evrogen) was added for RNA fixation and long-term storage at −80 °C. One month later, we determined the rats’ resistances to SHE at a critical altitude (11,500 m) once in a decompression chamber based on the “gasping time,” following which the animals assumed a lateral position and showed signs of asphyxia (Kirova et al., 2013; Dzhalilova et al., 2019). The rats were classified as tolerant (gasping time >240 s, n = 6) or susceptible (gasping time <80 s, n = 6) based on the SHE results. Animals with moderate tolerances (80–240 s, n = 18) were excluded from the rest of the study to focus on the most contrasting phenotypes. The selected animals were euthanized 1 month after SHE in the decompression chamber via intramuscular administration of Zoletil at a dose of 50 mg/kg. Death was confirmed by the complete cessation of breathing, absence of a palpable heartbeat, and loss of corneal reflex. For each animal, different researchers were involved during the procedures as follows: the first researcher (D.D.) modeled the SHE (this investigator was the only person aware of the group allocations); the second (M.M.) and third (I.T.) researchers were responsible for the euthanized animals; the fourth (M.K.) and fifth (V.K.) researchers conducted the polymerase chain reaction (PCR), morphological, and morphometric studies. No animal deaths were observed during the experiments, and none of the rats survived at the end of the experiments.

### 2.3 Sample collection

At the time of euthanization by intramuscular administration of Zoletil (50 mg/kg) 1 month after SHE, we collected 1 mL of venous blood from the jugular vein of each animal; the erythrocytes were then lysed and IntactRNA (Evrogen) was added immediately to the sample to fix the RNA before being stored at −80 °C. In addition, the lungs of the animals were collected for morphological and morphometric analyses, and these were fixed in Carnoy’s fluid for 2 h before processing.

### 2.4 Evaluation of mRNA expression in peripheral blood leukocytes

The total RNA was isolated from the leukocytes using the RNA Solo kit (Evrogen) containing the DNase rDSN, and reverse transcription was performed using the MMLV RT kit (Evrogen) in accordance with manufacturer instructions. The expression of *Hif1a*, *Epas1*, *Hif3a*, *Arnt*, *Vegf*, *Epo*, *Egln1*, *Nfkb*, *Il1b*, *Tnfa*, and *Tgfb* mRNAs in the leukocytes was determined from two technical replicates using the 5X qPCRmix-HS SYBR (Evrogen) and primers synthesized by Evrogen (Supplementary Table S1) via the real-time polymerase chain reaction (RT-PCR) method relative to the expression of *Gapdh* on a DTprime amplifier (DNA-Technology). The relative mRNA concentrations of the indicated genes were calculated by the ΔΔCq method (Livak and Schmittgen, 2001).

### 2.5 Evaluation of microRNA expression in peripheral blood leukocytes

MicroRNAs were isolated from the leukocytes using the SKYAmp miRcute miRNA Isolation kit (SkyGen), following which reverse transcription was performed with stem-loop primers (Supplementary Table S2) (Pritchard et al., 2012) and the MMLV RT kit (Evrogen) in accordance with manufacturer instructions. The expression of microRNAs *rno-miR-210-5p*, *rno-miR-210-3p*, *rno-miR-107-5p*, *rno-miR-107-3p*, *rno-miR-145-5p*, *rno-miR-145-3p*, *rno-miR-155-5p*, and *rno-miR-155-3p* in the leukocytes was determined from two technical replicates using the 5X qPCRmix-HS SYBR mixture (Evrogen) and primers synthesized by Evrogen (Supplementary Table S1) via the RT-PCR method relative to the expression of *SNORD61* on a DTprime amplifier (DNA-Technology). The relative microRNA concentrations of the indicated genes were calculated by the ΔΔCq method (Livak and Schmittgen, 2001).

### 2.6 Morphological and morphometric studies of the lungs

The lungs were embedded in paraffin, and histological sections were prepared by staining with hematoxylin and eosin. The neutrophils were counted in the interalveolar septa of the respiratory tract in 10 standard fields of view (25,000 μm^2^).

### 2.7 Statistical methods

Statistical processing of the obtained results was performed in Statistica 8.0 and GraphPad Prism 8.0. The distribution of the indicators was determined using the Kolmogorov–Smirnov criterion. Since the data were not normally distributed, the reliability of differences between the indicators was determined using the non-parametric Mann–Whitney (differences between animals with different tolerances to hypoxia), Kruskal–Wallis, and Dunn (differences between animals with different tolerances to hypoxia by taking into account the dynamics of changes) criteria. The data were expressed in terms of the median (Me) values and interquartile ranges (25%–75%). The differences were considered to be statistically significant at *p* < 0.05.

## 3 Results

### 3.1 Genes regulating cellular response to hypoxia expression in peripheral blood leukocytes

**FIGURE 2:**
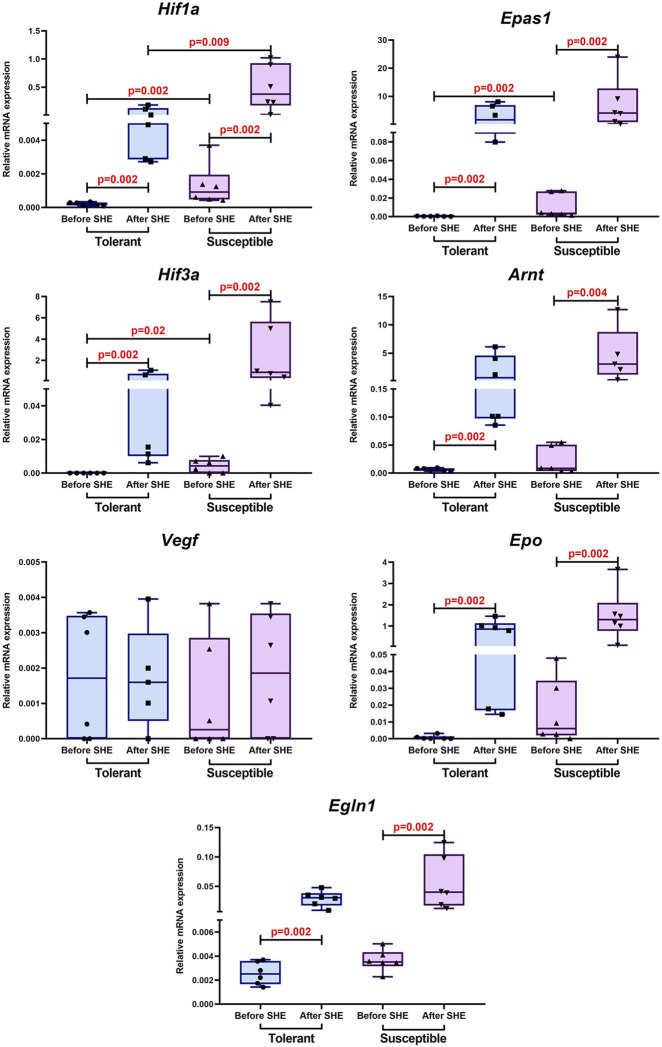
*Hif1a*, *Epas1*, *Hif3a*, *Arnt*, *Vegf*, *Epo*, and *Egln1* expression levels in the peripheral blood leukocytes of hypoxia-tolerant (n = 6) and hypoxia-susceptible (n = 6) rats before and 1 month after sublethal hypoxic exposure (SHE). The data are shown as median (Me) value with interquartile range (IQR: 25%–75%).

Before SHE, the *Hif1a*, *Epas1*, and *Hif3a* mRNA expression levels were statistically significantly higher in the hypoxia-susceptible animals than hypoxia-tolerant ones. In addition, to assess the effects of SHE on the animals, we determined the mRNA expression levels 1 month after loading; regardless of the initial hypoxia tolerance, the *Epas1*, *Hif3a*, *Arnt*, *Epo*, and *Egln1* expression levels increased. Furthermore, the *Hif1a* expression level increased after SHE, while this level was higher in the hypoxia-susceptible animals than the hypoxia-tolerant ones. No statistically significant changes were detected in the *Vegf* expression levels before and after SHE (Figure 2).

### 3.2 Expression of microRNAs regulating cellular response to hypoxia in peripheral blood leukocytes

**FIGURE 3:**
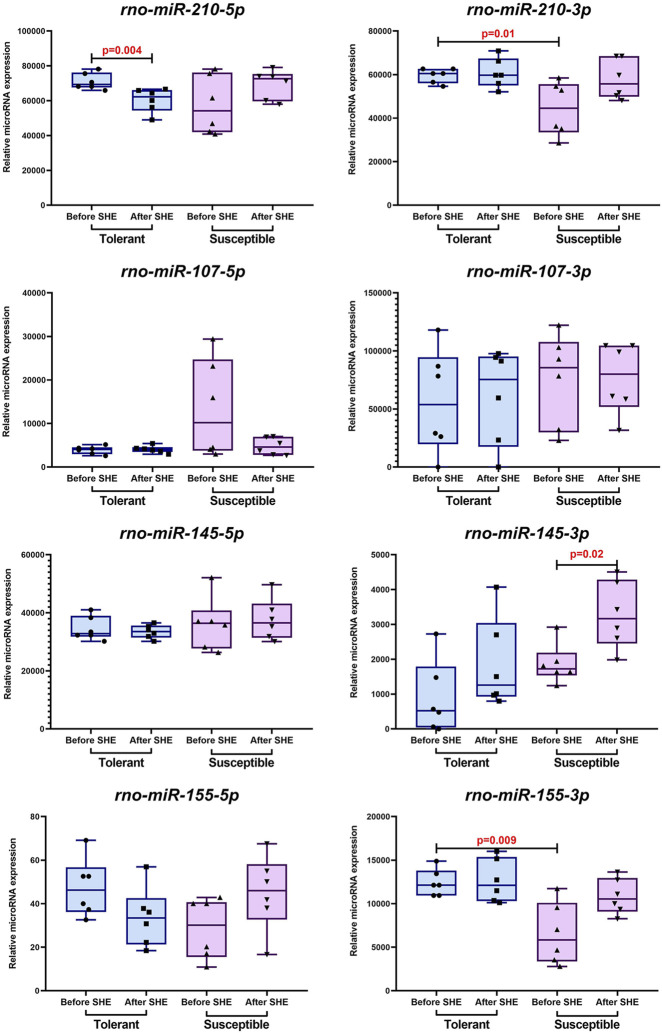
*Rno-miR-210-5p*, *rno-miR-210-3p*, *rno-miR-107-5p*, *rno-miR-107-3p*, *rno-miR-145-5p*, *rno-miR-145-3p*, *rno-miR-155-5p*, and *rno-miR-155-3p* expression levels in the peripheral blood leukocytes of hypoxia-tolerant (n = 6) and hypoxia-susceptible (n = 6) rats before and 1 month after SHE. The data are shown in terms of Me and IQR (25%–75%).

Before SHE, the *rno-miR-155-3p* and *rno-miR-210-3p* expression levels were lower in the hypoxia-susceptible animals than tolerant rats. At the same time, the *rno-miR-145-3p* expression level increased in the hypoxia-susceptible rats after SHE, while the tolerant animals demonstrated a decrease in *rno-miR-210-5p* expression. No statistically significant differences were observed in the expression levels of *rno-miR-107-5p*, *rno-miR-107-3p, rno-miR-145-5p*, and *rno-miR-155-5p* before and after SHE (Figure 3).

### 3.3 Expression of genes regulating cellular response to inflammation in peripheral blood leukocytes

**FIGURE 4:**
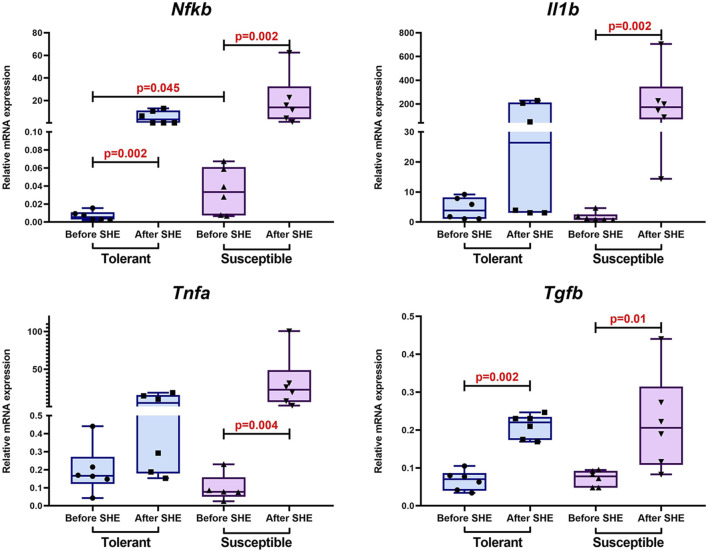
*Nfkb*, *Il1b*, *Tnfa*, and *Tgfb* expression levels in the peripheral blood leukocytes of hypoxia-tolerant (n = 6) and hypoxia-susceptible (n = 6) rats before and 1 month after SHE; the data are shown in terms of Me and IQR (25%–75%).

Before SHE, the *Nfkb* expression level in the peripheral blood leukocytes of animals susceptible to hypoxia was statistically significantly higher than that of tolerant animals, while no differences were detected in the expression levels of proinflammatory cytokine genes *Il1b* and *Tnfa* as well as anti-inflammatory cytokine gene *Tgfb*. After SHE, the *Nfkb* and *Tgfb* expression levels increased regardless of the initial hypoxia tolerance. At the same time, we detected increases in the expression levels of proinflammatory cytokine genes *Il1b* and *Tnfa* only in animals susceptible to oxygen deficiency (Figure 4).

### 3.4 Morphological and morphometric studies of the lungs

**FIGURE 5:**
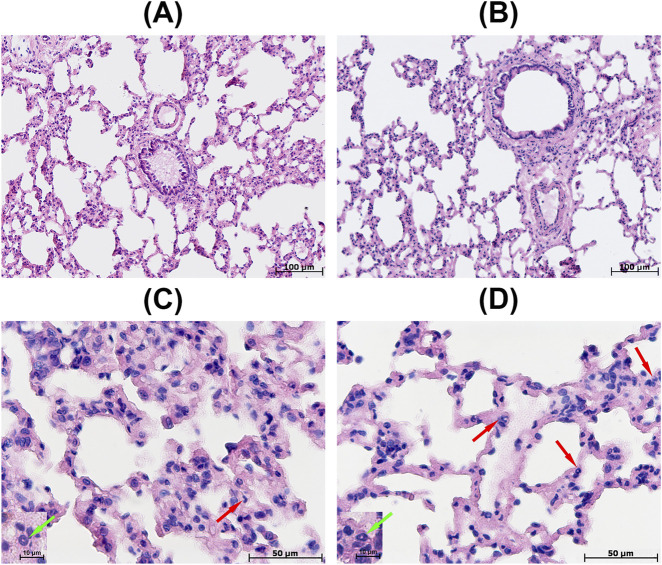
Morphological characteristics of the lungs in animals **(A,C)** tolerant and **(B,D)** susceptible to hypoxia after SHE. **(A)** Desquamated epithelial cells in the lumen of the bronchi; **(B)** widening of the perivascular gap; **(C,D)** neutrophils in the interalveolar septa (red arrows), with the inset images showing the magnified neutrophils (green arrows) based on hematoxylin and eosin staining.

Regardless of the initial hypoxia tolerance, the lung morphological study revealed granular eosinophilic contents and isolated desquamated epithelial cells in the lumen of second- and third-order bronchi (Figures 5A,B). The adventitial layer revealed mild lymphocyte and histiocyte infiltration; the bronchi-associated lymphoid tissue consisted of small lymphocyte clusters. In the respiratory region, the alveolar ducts and alveoli were free and wide, while the interalveolar septa were thin and contained isolated neutrophils (Figures 5C,D).

**FIGURE 6:**
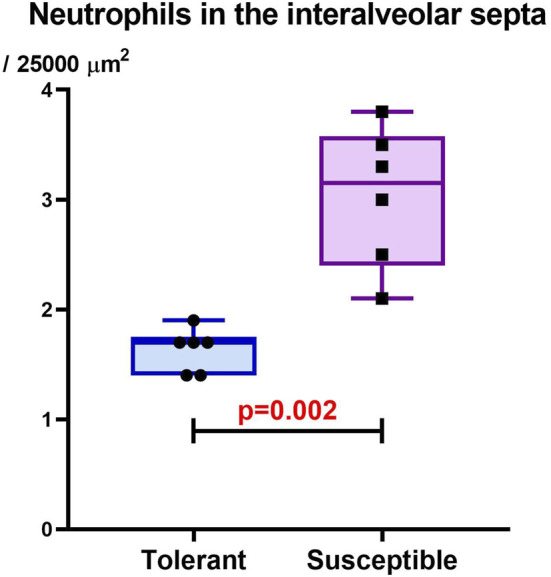
Neutrophil numbers in the interalveolar septa of the lungs in rats tolerant (n = 6) and susceptible (n = 6) to hypoxia 1 month after SHE; the data are shown in terms of Me and IQR (25%–75%).

The lung morphometric study showed that the neutrophil numbers in the interalveolar septa were higher in the hypoxia-susceptible rats than the tolerant ones (Figure 6).

## 4 Discussion

### 4.1 mRNA and microRNA expression levels as potential biomarkers of initial oxygen deficiency tolerance

**FIGURE 7:**
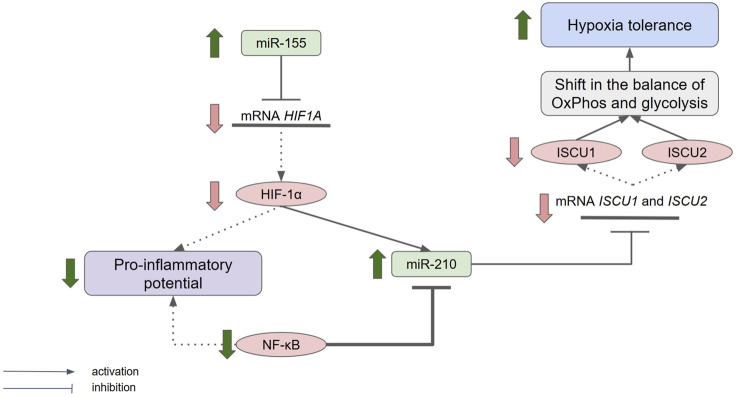
Schematic of the supposed hypoxia tolerance mechanism. HIF, hypoxia-inducible factor; NF-κB, nuclear factor kappa B; ISCU1 and ISCU2, iron–sulfur cluster assembly proteins; OxPhos, oxidative phosphorylation.

We found that the initial *Hif1a*, *Epas1*, and *Hif3a* levels and *Nfkb* mRNA expression were higher in the peripheral blood leukocytes of hypoxia-susceptible animals, while the *rno-miR-155-3p* and *rno-miR-210-3p* levels were higher in the hypoxia-tolerant rats. It is known that the mRNA transcript of the *HIF1A* gene includes binding sites for miR-155 in the 3′-untranslated region (Yang et al., 2016). Therefore, the high expression of *rno-miR-155-3p* in the hypoxia-tolerant animals before SHE likely indicates the degradation of this microRNA’s target mRNA, resulting in a lower *Hif1a* expression than that in susceptible animals. At the same time, the initial high expression levels of *Hif1a* and *Nfkb* in the hypoxia-susceptible rats may indicate a high proinflammatory potential in these animals given that the relationship between cellular response to hypoxia and inflammation, particularly the interactions of transcription factors NF-κB and HIF-1α, has been described in detail in literature (Rius et al., 2008; van Uden et al., 2008; 2011). It was also demonstrated that NF-κB can bind and inhibit the expression of *miR-210* (Zhou et al., 2024), whereas HIF-1α and HIF-2α can activate such expression (Moszyńska et al., 2022). According to the obtained data, the *rno-miR-210-3p* expression level in hypoxia-susceptible rats before SHE was lower than that in tolerant rats despite that higher level of *Hif1a* expression. This means that the NF-κB-dependent regulatory pathway probably has a more pronounced effect on this microRNA, which confirms the hypothesis of a high proinflammatory potential in hypoxia-susceptible animals. The targets of miR-210 are the 3′-untranslated regions of the iron-sulfur cluster assembly protein transcripts ISCU1 and ISCU2, which are mainly involved in ensuring electron transport and the redox reactions in the metabolism of various molecules (Rouault and Tong, 2008; Chan et al., 2009). The high level of *rno-miR-210-3p* expression in the hypoxia-tolerant rats may indicate suppression of ISCU1 and ISCU2 translation, which would result in decreased mitochondrial respiration and a shift in the balance between oxidative phosphorylation and glycolysis toward the latter, leading to the development of hypoxia tolerance (Figure 7).

According to literature, hypoxia tolerance is correlated with general physical fitness, the ability to inactivate toxins, the ability to adapt, and with the probable plasticity of innate immune responses (Pavlik et al., 2018; Mironova et al., 2019). In addition, it was demonstrated that the structural organization and functional mitochondrial activities differed among the cells of the cerebral cortex, liver, and heart in animals having different tolerances to oxygen deficiency (Pavlik et al., 2018; Mironova et al., 2019). The hypoxia-tolerant animals are characterized by high mitochondria counts with denser packing of the cristae, a darker matrix, a large number of small and functionally more active mitochondria, and higher concentrations of the mitochondrial enzymes compared to the hypoxia-susceptible rats. The phenotypic ultrastructural, functional, and metabolic differences identified by Pavlik et al. (2018) and Mironova et al. (2019) indicate greater activity of the respiratory chain in hypoxia-tolerant rats than susceptible ones (Pavlik et al., 2018; Mironova et al., 2019). These differences suggest that energy metabolism may be one of the factors determining individual hypoxia tolerance. However, these data were obtained by researchers after determining the hypoxia tolerances in a decompression chamber, which could have affected the results. In addition, our hypothesis about the initial shift in oxidative phosphorylation toward glycolysis in tolerant animals is based on assessing the mRNA and microRNA expression levels in the blood cells and not the protein content in tissues. Therefore, the identified contradiction may be associated with a different cellular composition, including lymphocyte subpopulations. This could reflect the earlier molecular stage in the development of hypoxia tolerance since changes to the transcriptome, proteome, and cell organization as well as associated structures occur at different points in time, implying that one cannot expect unambiguous correspondence (Haghighi et al., 2022).

### 4.2 Effects of SHE on the organism one month after loading

According to literature, the characteristics of hypoxia-tolerant and hypoxia-susceptible animals along with their resistances to oxygen deficiency are often studied in a decompression chamber, and the experiments themselves are carried out no earlier than 1 month after hypoxic exposure (Lukyanova and Kirova, 2011). However, it was previously demonstrated that determining resistance to oxygen deficiency through SHE in a decompression chamber has an immunomodulatory effect on the cytokines production by blood cells, which persists even a month after exposure (Dzhalilova et al., 2024). In our study, we found that the *Epas1*, *Hif3a*, *Arnt*, *Epo*, *Egln1*, *Nfkb*, and *Tgfb* expression levels increased 1 month after SHE regardless of resistance to oxygen deficiency; however, *Il1b* and *Tnfa* expression was found to have increased only in the hypoxia-susceptible rats. In addition, *Hif1a* expression increased and was higher in the hypoxia-susceptible rats than tolerant ones. We also noted increased *rno-miR-145-3p* expression in the hypoxia-susceptible rats and decreased *rno-miR-210-5p* expression in the hypoxia-tolerant rats.

The increased expression of genes encoding three isoforms of the HIF-α subunit and HIF-1β protein after SHE is associated with responses to hypoxic stress as well as increased expression of one of the target genes *Epo* (Keith et al., 2011). Moreover, increased expression of *Egln1* is detected regardless of resistance to oxygen deficiency; this gene encodes PHD2, which can induce proteasomal degradation of the HIF-α subunits (Haase, 2017). Perhaps, the feedback loop is activated to reduce the content of HIF-α proteins to reduce the severity of the inflammatory responses.

MicroRNAs are known to be involved in modulating the activities of the HIF-α subunits. The increase in *rno-miR-145-3p* expression in animals susceptible to hypoxia may possibly be associated with inhibition of the HIF-2α-mediated response (Zhang et al., 2014). According to literature, HIF-1α regulates the response to acute oxygen deficiency, while HIF-2α is activated during prolonged hypoxia (Loboda et al., 2010; Moszyńska et al., 2022). The effects of SHE on animals susceptible to hypoxia lasted less than 80 s, which corresponds to acute hypoxia.

Another target of miR-145 is the SIRT1 transcript, whose inhibition promotes NF-κB protein expression and synthesis leading to activation of the autophagy molecule Beclin-1 and development of severe lung injury (Dai et al., 2021; Ke et al., 2023). It was demonstrated that miR-145 overexpression in miR-145Tg transgenic mice (Sawant et al., 2020) is accompanied by an increase in the number of immune cells in the lungs, indicating leukocyte extravasation (Thomas et al., 2021) as a process of transendothelial migration from the vascular lumen to inflammatory foci in tissues (Weber et al., 2007). In our study, the *rno-miR-145-3p* expression level as well as neutrophil numbers in the interalveolar septa of the lungs were higher in the hypoxia-susceptible rats, indicating the development of more pronounced proinflammatory responses compared to those observed in the tolerant animals.

The *Il1b* and *Tnfa* gene expression after SHE increased only in animals susceptible to hypoxia, which probably indicate activation of proinflammatory responses in these rats (Malkov et al., 2021). Further, in response to the increased *Nfkb* expression in hypoxia-tolerant animals compared to the susceptible ones, we observed an increase in the anti-inflammatory *Tgfb* cytokine rather than the proinflammatory *Il1b* and *Tnfa* cytokines, indicating activation of negative feedback to inhibit NF-κB-dependent proinflammatory responses and consequently higher rates of adaptation to hypoxia. A decrease in the *rno-miR-210-5p* expression level in hypoxia-tolerant animals may be caused by increased gene expression and NF-κB protein synthesis in these animals, which act as negative regulators (Zhou et al., 2024). At the same time, there were no detectable changes in the expression of *rno-miR-210-5p* and *rno-miR-210-3p* in animals susceptible to hypoxia, which may be associated with an earlier stage of adaptation to low oxygen compared to the hypoxia-tolerant animals.

Our data indicate that hypoxia-susceptible rats are predisposed to exacerbated proinflammatory responses following SHE owing to their higher initial proinflammatory potential. This distinct physiological trait is a probable factor in the increased severity of inflammatory and tumor-based diseases observed in these animals, which would designate them as a high-risk population. However, the question of whether the primary factor is hypoxia tolerance or proinflammatory potential remains unresolved. It is known that the HIF signaling pathway emerged over 600 million years ago in the earliest multicellular organisms (*Trichoplax adhaerens*) (Loenarz et al., 2011), whereas homologs of NF-κB and associated proinflammatory mechanisms appeared later (approximately 500 million years ago) in arthropods (*Limulus*) (Wang et al., 2006). Thus, the hypoxia response system is probably the primary factor in the evolutionary sense, and the initial hypoxia tolerance likely determines the baseline proinflammatory potential rather than vice versa. However, there are insufficient data in literature to either support or refute this hypothesis, necessitating further investigations into the evolutionary origins of both hypoxic and immune responses.

### 4.3 Study limitations and future perspectives

The present study has several limitations: the study did not include an intact group of control animals, so it is not possible to separate the SHE-related changes from age-related or other characteristics of the animals. Although the connection between HIF-1α and microRNAs is described in detail, the interaction mechanisms between HIF-2α and HIF-3α as well as HIF-1β and the microRNAs are not fully understood. For a comprehensive assessment of the initial hypoxia tolerance, additional studies are required that would include proteomic profiling as well as evaluations of the proinflammatory and anti-inflammatory cytokines and oxidative stress levels. Studying the ultrastructural features of the mitochondria as one of the main organelles that ensure metabolism under normoxic and hypoxic conditions in tolerant and susceptible animals can also strengthen this research and allow us to confirm or refute the mechanism of initial resistance to oxygen deficiency suggested herein. However, despite the limitations of this study, we investigated differences in the expression levels of mRNAs and microRNAs both before and after hypoxic exposure. This indicates that sublethal exposure to hypoxia in a decompression chamber when determining hypoxia tolerance has an extended effect on the organism that lasts at least a month. Thus, the indicators identified herein, which differ between the animals tolerant and susceptible to oxygen deficiency before SHE, can be considered as potential biomarkers of initial hypoxia tolerance. In the future, this will allow the development of a panel of hypoxia resistance markers and investigations that take into account individual responses to oxygen deficiency without prior exposure to hypoxia. Understanding the relationships between the HIFs, hypoxia tolerance, and regulatory roles of microRNAs are necessary for developing new approaches to personalized therapy for diseases accompanied by oxygen deficiency, including systemic inflammation.

## 5 Conclusion

In this study, compared to hypoxia-tolerant rats, hypoxia-susceptible Wistar rats demonstrated an initially high proinflammatory potential characterized by high levels of *Hif1a*, *Epas1*, *Hif3a*, and *Nfkb* mRNA expression along with low levels of *rno-miR-155-3p* and *rno-miR-210-3p* microRNAs in the peripheral blood leukocytes. In both hypoxia-tolerant and hypoxia-susceptible animals, we observed the activation of proinflammatory responses against oxygen deficiency, which persisted for at least a month after SHE; however, in animals susceptible to hypoxia, these responses were combined with increased expression of the proinflammatory cytokines *Il1b* and *Tnfa*. Thus, hypoxia-susceptible rats were found to have an initial high proinflammatory potential leading to more pronounced proinflammatory responses after SHE compared to the hypoxia-tolerant animals; this may indicate a more severe course of inflammatory and tumor-based diseases in such animals, thereby allowing us to characterize them as a risk group. The data obtained herein are expected to contribute to the development of new approaches to determining resistance to oxygen deficiency without hypoxic stress in both laboratory animals and humans.

## Data Availability

The original contributions presented in the study are included in the article/Supplementary Material, and any further inquiries may be directed to the corresponding author.
