# Supplementary material for: MicroRNAs and genes regulating responses to hypoxia and inflammation expression levels in blood leukocytes as potential biomarkers of initial oxygen deficiency tolerance
Source: Front Mol Biosci. 2026 Apr 1;13:1796967. doi: 10.3389/fmolb.2026.1796967 (PMC13079197; doi:10.3389/fmolb.2026.1796967)
Supplement: Supplementary file 1 [file DataSheet1.pdf]

Supplementary Material

1 Supplementary Table S1. Oligonucleotide sequences for PCR

Gene		Sequences 5'→3'
Gapdh	forward	GCCAGCCTCGTCTCATAGAC
	reverse	CTTGCCGTGGGTAGAGTCAT
Hif1a	forward	GAGCCTTAACCTATCTGTCA
	reverse	CACAATCGTAACTGGTCAGC
Epas1	forward	AACCTTAAGTCGGCCACCTG
	reverse	TTGCTGTCCAAGGGGATGTC
Hif3a	forward	GAGGCTATCTGTGGACTCAGACT
	reverse	CGAGTATGTTGCTCCGTTTG
Arnt	forward	CGGGCAATACATCCACTGACG
	reverse	CATACACCACTCGGCCAGTC
Egln1	forward	GCATGAACAAGCACGGCAT
	reverse	GCCCTCGATCCAGGTGATC
Vegf	forward	CACGTTGGCTCACTTCCAG
	reverse	CAGGCTCCTGATTCTTCCAG
Epo	forward	CCGTCCCAGATACCAAAGTCA
	reverse	GGCGACATCAATTCCTTCTGA
Nfkb	forward	GACGATCCTTTCGGAACTG
	reverse	GCATATGCCGTCCTCACAG
Il1b	forward	TTCGACAGTGAGGAGAATGAC
	reverse	CGTCATCATCCCACGAGTCA
Tnfa	forward	GTTCCGTCCCTCTCATACACTG
	reverse	GAAGTTCAGTAGACAGAAGAGCGT
Tgfb	forward	CGTGGCTTCTAGTGCTGACG
	reverse	TGGCGAGCCTTAGTTTGGAC
SNORD61	forward	CCCCCCGCTATGATGAATTTGATTGCAT
	reverse	CCCCAAGCTCAGAACTTCTTAGAGGACAA
rno-miR-210-5p	forward	GTCACACGACACCCGTCACCGA

rno-miR-210-3p	forward	AGTCGGCGACAGTGTGCGTGTC
rno-miR-107-5p	forward	CCCTGTTCCGTTGTGACATTTCTTCGA
rno-miR-107-3p	forward	CCCACTATCGGGACATGTTACGACGA
rno-miR-145-5p	forward	CCCTCCCTAAGGACCCTTTTGACCTG
rno-miR-145-3p	forward	CCCCCCTTGTCATAAAGGTCCTTAGG
rno-miR-155-5p	forward	CCCCCTGGGGATAGTGTTAATCGTAATT
rno-miR-155-3p	forward	CCCCCAATTACGATTGTCCATCCTC
rno-miR	reverse	TCCAGTGCAGGGTCCGAGGTATTC

2 Supplementary Table S2. Oligonucleotide sequence for RT for microRNA

microRNA	«stem-loop» primers sequences 5’→3’
rno-miR-210-5p	GTCGTATCCAGTGCAGGGTCCGAGGTATTCGCACTGGATACGACTCGGTG
rno-miR-210-3p	GTCGTATCCAGTGCAGGGTCCGAGGTATTCGCACTGGATACGACGACACG
rno-miR-107-5p	GTCGTATCCAGTGCAGGGTCCGAGGTATTCGCACTGGATACGACTCGAAG
rno-miR-107-3p	GTCGTATCCAGTGCAGGGTCCGAGGTATTCGCACTGGATACGACTCGTCG
rno-miR-145-5p	GTCGTATCCAGTGCAGGGTCCGAGGTATTCGCACTGGATACGACCAGGTC
rno-miR-145-3p	GTCGTATCCAGTGCAGGGTCCGAGGTATTCGCACTGGATACGACCCTAAG
rno_miR-155_5p	GTCGTATCCAGTGCAGGGTCCGAGGTATTCGCACTGGATACGACAATTAC
rno-miR-155-3p	GTCGTATCCAGTGCAGGGTCCGAGGTATTCGCACTGGATACGACGAGGAT
